# Supplementary material for: Collapse and rescue of cooperation in evolving dynamic networks
Source: Nat Commun. 2018 Jul 12;9:2692. doi: 10.1038/s41467-018-05130-7 (PMC6043585; doi:10.1038/s41467-018-05130-7)
Supplement: Supplementary file 1 — Supplementary Information [file 41467_2018_5130_MOESM1_ESM.pdf]

Supplementary Information

Collapse and rescue of cooperation in evolving dynamic networks

Erol Akçay

eakcay@sas.upenn.edu

Supplementary Note 1: Supplementary figures for the coauthor game

Assortment and mean degree in networks with fixed p_n and p_r

Supplementary Figure 1 shows the expected relatedness under neutrality as a function of the (fixed) linking probabilities p_n and p_r. Supplementary Figure 2 shows the relationship between mean degree of networks for fixed linking probabilities, for the same simulations as in Figure 1.

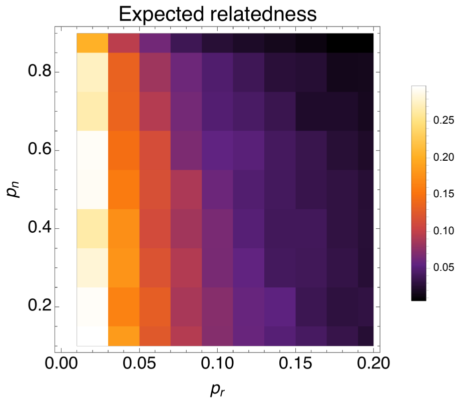

Supplementary Figure 1: Expected relatedness (calculated as the regression coefficient between the cooperation types of connected individuals) in neutrally evolving networks as a function of p_n and p_r. As can be seen in the figure, relatedness decreases with increasing p_r, and at high values of p_n, explaining the patterns we observe for cooperation. For each value of linking probabilities, networks are run for 2000 time steps and the regression on the resulting network is averaged over 100 replicates. $N = 100$, $\mu = 0.001$.

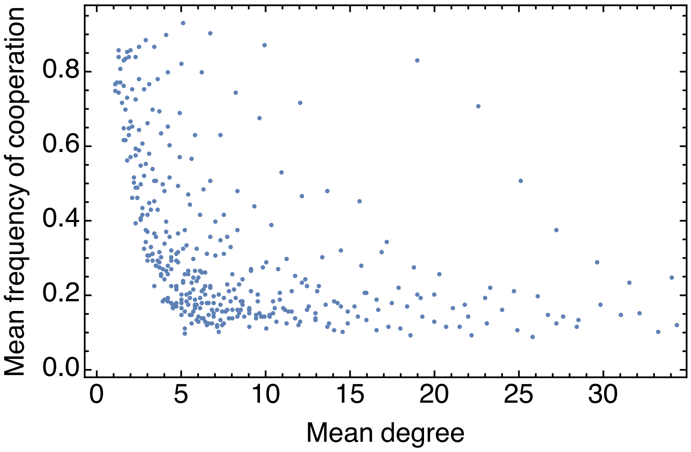

Supplementary Figure 2: Mean frequency of cooperation vs mean degree in networks with fixed linking traits, for the same simulations as in Figure 1. As the figure shows, there is a general negative relationship between degree and the frequency of cooperation, but the mean degree of a network does not uniquely fix the frequency of cooperation.

Results with larger network sizes

Supplementary Figures 3 and 4 depict results with evolving networks for network sizes $N = 200$ and $N = 500$, respectively. They show that long-term average evolutionary patterns remain unchanged with network size. Supplementary Figure 5 plots the mean degree in evolved networks with synergistic benefits (same setting as Figure 5 in the main text). In section A below, I discuss results that look at polymorphisms within populations in larger networks.

Results under strong selection

In the main text, I present results under relatively weak selection ($\delta = 0.1$), where the social trait does not directly affect the social structure (but does affect it through the evolutionary feedback on structuring traits). Here, I present results with stronger selection $\delta = 0.5$), where the network structure is directly affected by the presence or absence of cooperation on the network.

The main patterns for the co-evolution of cooperation and linking traits under

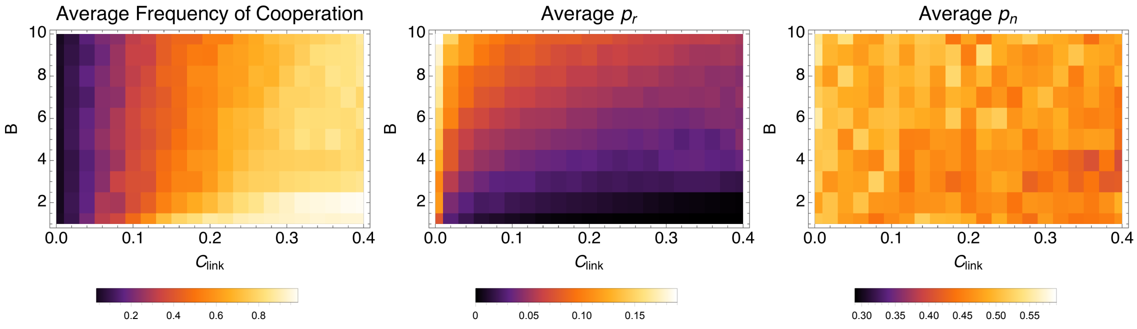

(a) (b) (c)

Supplementary Figure 3: Mean frequency of cooperation (a), p_r (b) and p_n (c) for larger networks $N = 200$. As in figure 4, each simulation was initiated with $p_r = 0.0001$, $p_n = 0.5$, frequency of cooperation at 0.5, and run for 10^5 generations. Values shown are means across the last 80,000 generations (in this case, 1.6×10^7 time steps). Other parameters values are $C = 0.5$, $D = 0$, $\mu = \mu_l = 0.01$, $\delta = 0.5$, $\sigma_n = \sigma_r = 0.01$.

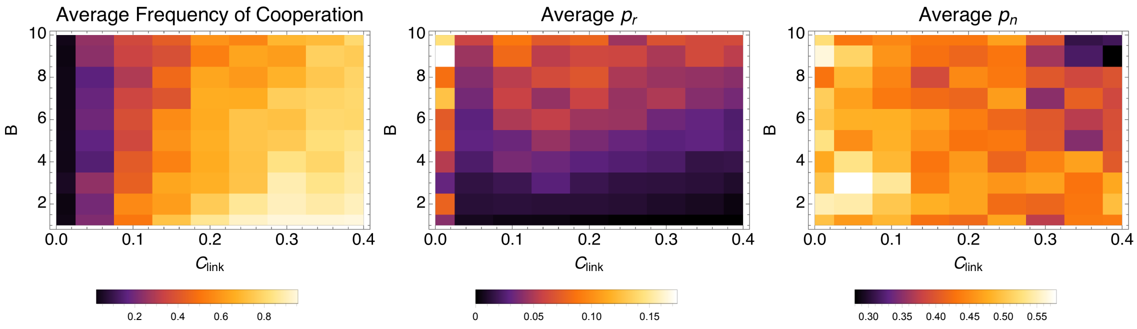

(a) (b) (c)

Supplementary Figure 4: Mean frequency of cooperation (a), p_r (b) and p_n (c) for larger networks with $N = 500$. Simulations are run as in Supplementary Figure 3, except for 2×10^4 generations. Values shown are means across the last 10^4 generations (5×10^6 time steps). Other parameters values are $C = 0.5$, $D = 0$, $\mu = \mu_l = 0.01$, $\delta = 0.5$, $\sigma_n = \sigma_r = 0.01$.

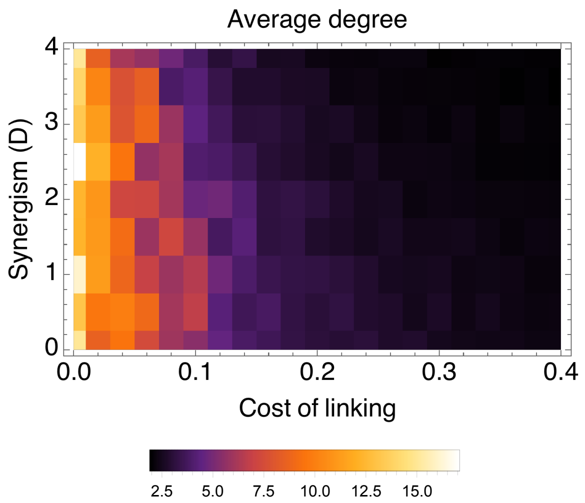

Supplementary Figure 5: Mean degree of evolved networks with synergistic benefits, for the same simulations as in Figure 5 in the main text, showing that when cooperation is maintained with synergistic benefits and linking costs, population evolve to be very sparsely connected.

strong selection are similar to under weak selection. However, under strong selection, social inheritance experiences more directional selection, and a somewhat unexpected pattern emerges. In particular, p_n initially decreases as B increases from low values (Supplementary Figure 6(c); see also Supplementary Figure 7 for a finer resolution look at this region). This is caused by a subtle parent-offspring conflict over social inheritance. In a cooperative population, offspring inheriting links is costly for the parent, as the benefit the parent receives from its connections will get diluted, in addition to receiving less benefit from the offspring. As B increases, the resolution of this conflict tends to favor the parents, since in a cooperative population and strong selection, only the highest-degree individuals tend to produce, and over time accumulate even more degrees. Thus, parents on average have much higher reproductive value than newborns, and therefore the conflict is resolved in their favor, with lower p_n. With further increasing benefit, p_r increases, and the dilution effect gets less important (since it scales as $1/d$), which shifts the resolution back towards the offspring's favor.

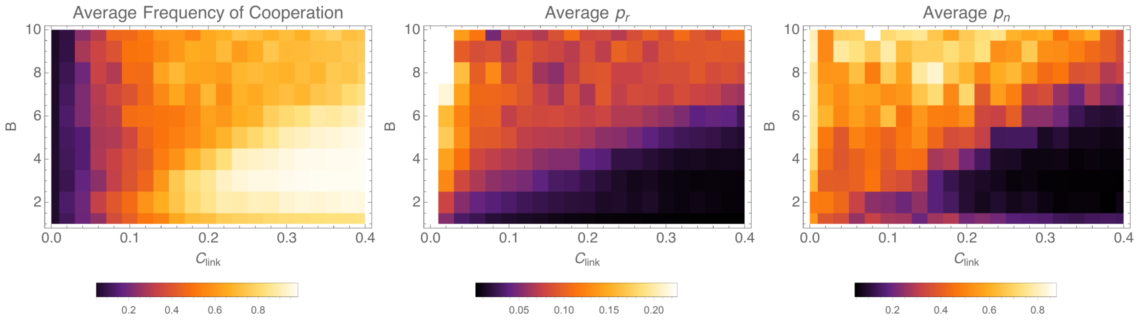

(a) (b) (c)

Supplementary Figure 6: Coevolution of cooperation and linkint traits under strong selection. Mean frequency of cooperation (a), p_r (b) and p_n (c) over 10 replicate simulations, averaged across time, as a function of the benefit from cooperation and the cost of linking. Each simulation was initiated with $p_r = 0.0001$, $p_n = 0.5$, frequency of cooperation at 0.5, and run for 10^5 generations. The first 2×10^4 were discarded to capture the steady-state of the stochastic dynamics, so that the shown averages are means across the 80,000 generations. Parameter values are $N = 100$, $C = 0.5$, $D = 0$, $\mu = \mu_l = 0.01$, $\delta = 0.5$, $\sigma_n = \sigma_r = 0.01$.

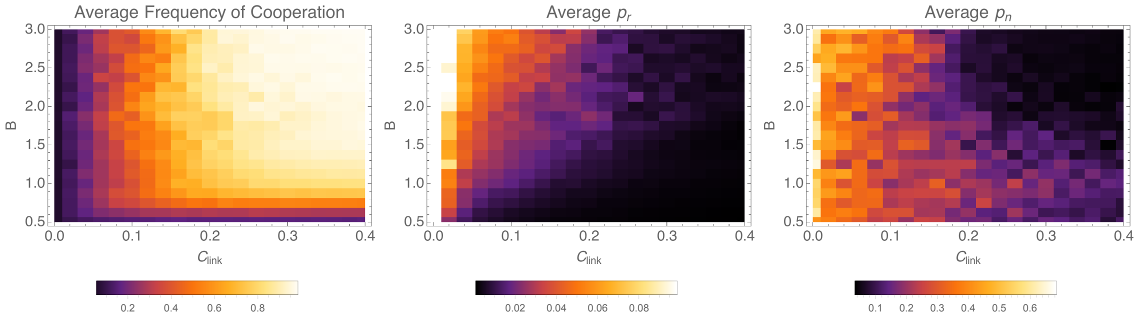

(a) (b) (c)

Supplementary Figure 7: A finer-scale look at the mean frequency of cooperation (a), p_r (b) and p_n (c) at low values of the benefit B under strong selection. As in Supplementary Figure 6, each simulation was initiated with $p_r = 0.0001$, $p_n = 0.5$, frequency of cooperation at 0.5, and run for 10^5 generations. Values shown are means across the 80,000 generations. Parameter values are $N = 100$, $C = 0.5$, $D = 0$, $\mu = \mu_l = 0.01$, $\delta = 0.5$, $\sigma_n = \sigma_r = 0.01$.

Apperance of high social inheritance under strong selection

As Supplementary Figure 4c above shows, under strong selection p_n evolves to be low when cooperation is selected for. This would appear to be inconsistent with observed values of high p_n. It is possible that the strong selection case, where there is high skew with one or a few highly connected individuals doing most of the reproduction, does not accurately reflect natural populations. An alternative explanation lies in the fact that Ilany and Akçay[1] assume a neutrally evolving population, whereas strong selection changes the network structure. As shown in Supplementary Figure 8, a given "true" value of p_n with strong selection generally results in networks where a neutral model would infer higher values of p_n.

Evolution in large networks: polymorphisms and cycling

In the above, I focused on the long-term average frequency for cooperation and linking probabilities across different costs and benefit levels. These averages are informative for small population sizes, where due to drift the population will spend most of its time fixed (or almost fixed) for one type or the other, thus quantities averaged over longer time-periods are more informative. In larger populations, the population composition is less affected by drift, and therefore becomes more informative. Simulations in larger networks show that cooperators and defectors can co-occur persistently (though not necessarily stably), or cycle through phases of high cooperation with low p_r and low cooperation with high p_r, as shown in Supplementary Figure 9. The two regimes can happen in the same population evolving under the same parameters at different times.

When cooperators and defectors co-occur, they experience diverging selection pressures on the linking traits (Supplementary Figure 10). Cooperators are selected to minimize both inherited and random connections, while defectors are selected to increase their connection rates, especially the random linking probabilities. This divergence requires cooperators to be relatively common in the population, which happens when linking is costly, so that random connections are likely to provide a benefit. However, as defectors increasingly make more random con-

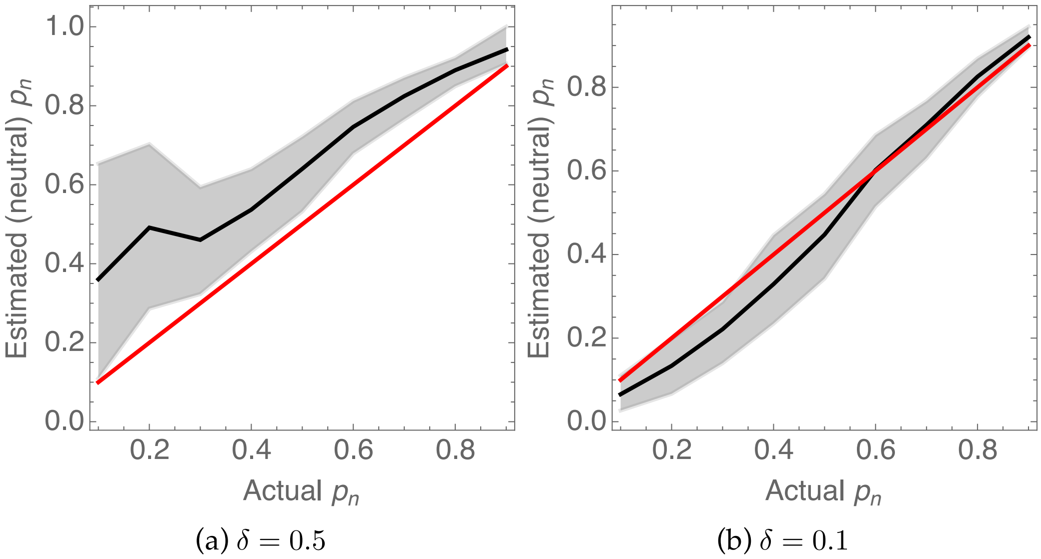

Supplementary Figure 8: Strong selection due to cooperation and high benefits creates the appearance of higher social inheritance than would be inferred under neutrality. In both panels, the black line depicts mean values of estimated p_n from 100 replicate networks, while the gray region shows the 90% confidence intervals and the red line estimated p_n = actual p_n. I simulated 100 replicate networks using $B = 4$, $C = 0.5$, $N = 100$, $p_r = 0.01$, $\mu = 0.01$, and p_n varying between 0.1 and 0.9. At the end of 20 generations, I sampled the network, calculating its mean degree and local clustering coefficients. I used the analytical expressions for the mean degree and clustering coefficients from Ilany and Akçay[1] to estimate the p_n and p_r coefficients that would produce these mean values under neutral dynamics. Panel (a) shows that networks under strong selection appear to have significantly higher neutral p_n estimates than their actual p_n. This effect disappears with relatively weak selection (Panel b).

nections, the benefit each can get from a given cooperative connection decreases due to the dilution of benefits in the coauthor game. This continues until the average degree of cooperators becomes too high, and the benefit from them too diluted, to compensate for the cost of linking, at which point the highly connected defector lineage goes extinct (Supplementary Figure 10). This pattern is especially apparent with moderate benefits and intermediate linking costs (as in Supplementary Figure 10), where cooperators can be maintained at high frequency. Under such conditions, the cycle of a minority fraction of defectors with increasing linking probabilities until they go extinct can repeat itself.

Supplementary Note 2: Results with the Prisoner's Dilemma game

In this section, I describe the same dynamical network model with a different payoff structure. In particular, instead of cooperators providing a fixed benefit that gets divided between all their connections and paying a fixed cost regardless of their degree, I assume that each cooperator provides a fixed per connection benefit, and pay a fixed cost per connection. In other words, the payoff to an individual is now:

$$u_i(t) = 1 + \sum_{j \neq i} p_j a_{ij} \left(B + p_i D\right) - p_i d_i(t) C - d_i(t) C_{\text{link}} \,, \tag{1}$$

Otherwise, the model works as described in the main text. One feature of the payoff function (1) is that the cost of cooperation and costs of linking work in exactly the same way. As shown below, that means that costs of linking cannot rescue cooperation of this kind.

Fixed linking probabilities

First, I keep p_n and p_r fixed and look at the long-term frequency of cooperation. Supplementary Figure 11 shows the results for strong selection (results are similar for weak selection). As in the coauthor game, cooperation evolves when p_r

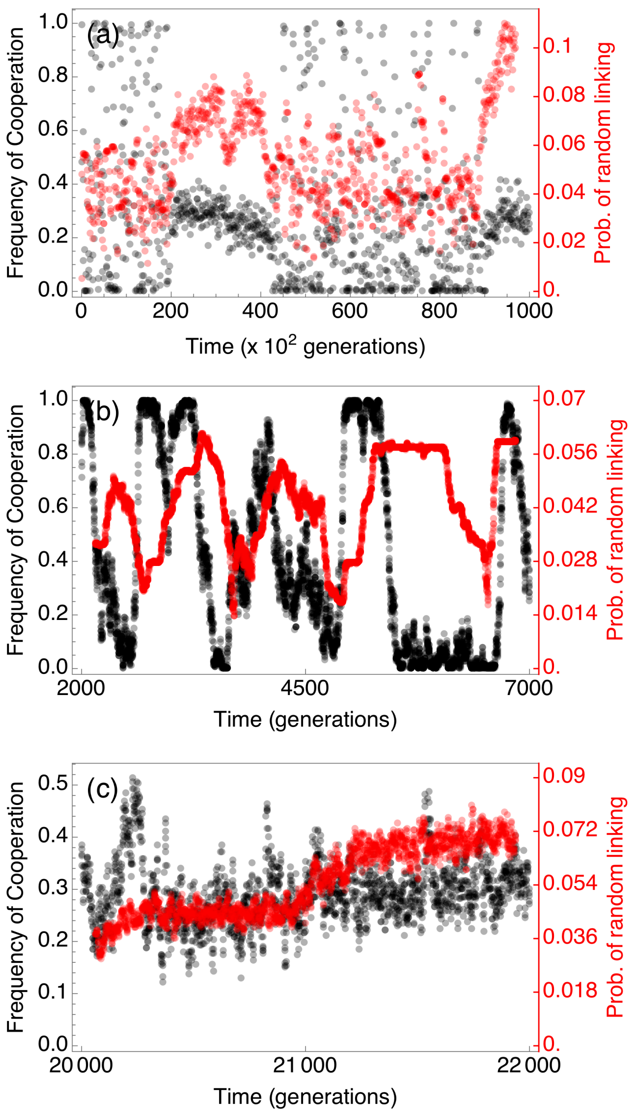

Supplementary Figure 9: Sample simulation illustrating the possibility of both cycling and persistent polymorphisms in larger networks. In all panels, the frequency of cooperation is in black, whereas the mean probability of random linking is in red. Panel (a) shows the time trajectory for 10^5 generations (i.e., 5×10^7 time steps), sampled at 100 generation intervals. Panel (b) and (c) show detailed trajectories for periods in the same simulation where the population is cycling vs. maintained as a polymorphism, respectively, recorded every generation. When the population is cycling, it maintains low variance in p_r, the mean value of which increases when cooperation is high, and decreases. Conversely, when the population is polymorphic, p_r has higher variance, due to defectors and cooperators having different values of p_r (see also Supplementary Figure 10). For this simulation run $N = 500$, $B = 10$, $C = 0.5$, $D = 0$, $C_{\text{link}} = 0.1$, $\delta = 0.1$, $\mu = \mu_l = 0.001$, $\sigma_n = \sigma_r = 0.01$.

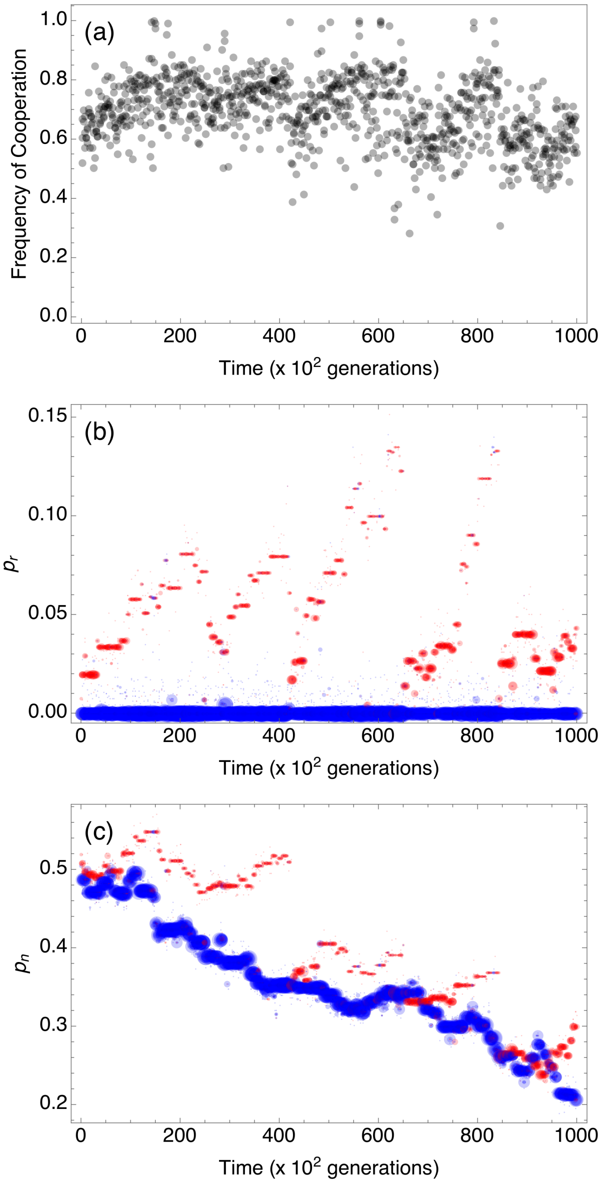

Supplementary Figure 10: Trajectories of frequency of cooperation (a), p_r (b), and p_n (c) in a larger population ($N = 500$) where a polymorphism can be maintained between cooperation and defection. In panels (b) and (c), blue color denotes cooperators, red defectors, and the size of the points is proportional to the frequency of that particular cooperation-linking probability genotype. Here, $B = 2$ and $C_{\text{link}} = 0.2$, which is a condition that supports a high average frequency of cooperation. The simulation was run for 10^5 generations (i.e., 5×10^7 time steps), with the population distribution recorded every 100 generations. It shows that in polymorphic populations, cooperators and defectors evolve divergent linking traits: while cooperators stably evolve low p_r and p_n values, defectors evolve higher p_r and to some extent, p_n values. However, increasing p_r is associated with the extinction of defectors, followed by their reemergence by mutation from the standing cooperator population with low p_r and p_n values, at which point the cycle repeats itself.

is low. On the other hand, somewhat differently from the coauthor game in the main text, p_n has a non-monotonic effect on cooperation: both low and high values of p_n select against cooperation compared to intermediate values. This is because unlike the coauthor game, the benefits from a cooperative partner do not get diluted over all the connections of the partner, and low p_n reduces the opportunity for cooperators to form mutually cooperating clusters. Therefore, forming more connected clusters favors cooperation, until the network becomes too connected. Incidentally, these results are directly comparable with those of Cavaliere et al.[2], who consider a model with $p_r = 0$ and vary p_n and the probability to connecting to the parent. Thus, their model with probability of connecting to the parent corresponds to the left-hand boundaries in Supplementary Figure 11. Thus, my results are consistent with the observation of Cavaliere et al. that cooperation persist in the population most of the time as long as p_n is not too high.

Evolving linking probabilities

Next, I let the linking probabilities co-evolve, as with the coauthor game in the main text, with the possibility of costly linking $C_{\text{link}} > 0$. As Supplementary Figure 12 shows, the self-limitation of cooperation is present again here. Moreover, we observe that costs of linking do not rescue cooperation. The reason can be seen in the payoff function (1): the linking costs just add an additional cost that is constant per link, exactly as the costs of cooperation for the prisoner's dilemma game (with the difference that they are paid by all individuals). That means if cooperation is favored by an initial configuration (say, with low p_r and intermediate p_n), it means that the costs of linking is low enough to be overcome by the benefits. In that case, selection will always favor higher linking probabilities (specifically, higher p_r), which will bring about a population structure where cooperation cannot persist anymore. Overcoming this feedback in the Prisoner's Dilemma therefore requires mechanisms other than costly linking (e.g., partner choice, or synergistic payoffs due to reciprocity).

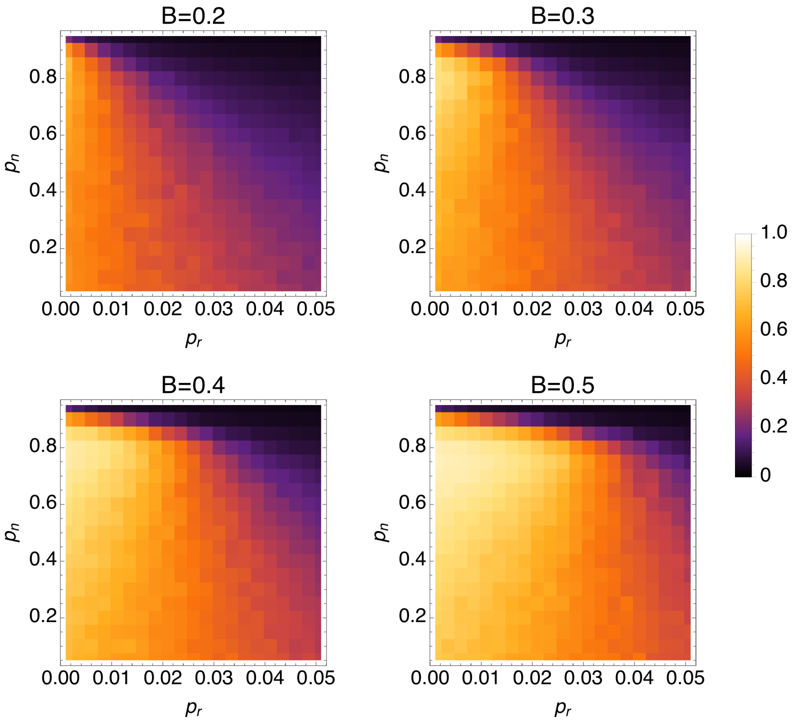

Supplementary Figure 11: Long-term frequency of cooperation for fixed values of p_n and p_r with weak selection ($\delta = 0.1$), $C = 0.1$, and B as given at the top of each panel. Simulations are run as in Figure 1: 100 replicate populations of 100 individuals were simulated for 500 generations (50,000 time steps) and the average frequency of cooperation in the last 400 generations are calculated. Mutation rate $\mu = 0.01$.

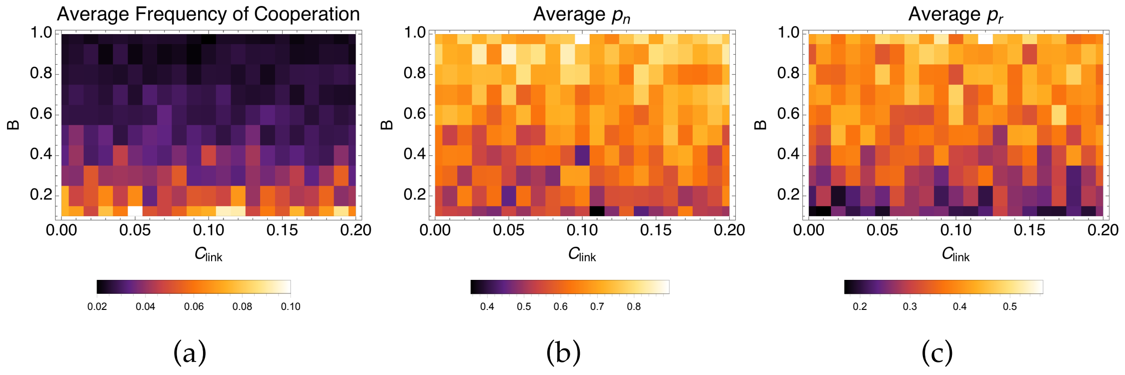

Supplementary Figure 12: Long-term average frequency of cooperation, p_n, and p_r with evolving linking probabilities, with weak selection ($\delta = 0.1$). Other parameters: $C = 0.1$, $D = 0$, $\mu = \mu_l = 0.01$, $\sigma_n = \sigma_r = 0.01$.

Supplementary references

[1] Ilany, A. & Akçay, E. Social inheritance can explain the structure of animal societies. *Nature Communications* **7**, 12084 (2016).

[2] Cavaliere, M., Sedwards, S., Tarnita, C. E., Nowak, M. A. & Csikász-Nagy, A. Prosperity is associated with instability in dynamical networks. *Journal of theoretical biology* **299**, 126–138 (2012).
